# In situ photografting during direct laser writing in thermoplastic microchannels

**DOI:** 10.1038/s41598-021-90571-2

**Published:** 2021-05-26

**Authors:** Jung Y. Han, Sarah Warshawsky, Don L. DeVoe

**Affiliations:** 1 Department of Mechanical Engineering, University of Maryland, College Park, 20742 USA; 2 Robert E. Fischell Institute for Biomedical Devices, University of Maryland, College Park, 20742 USA

**Keywords:** Fluidics, Nanofluidics, Photochemistry

## Abstract

A method for in situ photografting during direct laser writing by two-photon polymerization is presented. The technique serves as a powerful approach to the formation of covalent bonds between 3D photoresist structures and thermoplastic surfaces. By leveraging the same laser for both pattern generation and localized surface reactions, crosslinking between the bulk photoresist and thermoplastic surface is achieved during polymerization. When applied to in-channel direct laser writing for microfluidic device fabrication, the process yields exceptionally strong adhesion and robust bond interfaces that can withstand pressure gradients as high as 7 MPa through proper channel design, photoinitiator selection, and processing conditions.

## Introduction

Direct laser writing (DLW) offers a powerful path to fabricating high resolution 3-dimensional structures at the microscale. By taking advantage of multiphoton absorption within the laser focus, complex 3D patterns can be realized with resolution on the order of several hundred nanometers, and with centimeter scale work volumes achieved through the use of optical or motorized translation stages. This flexible nanolithographic technique has been used to demonstrate various elements with critical features below the optical diffraction limit^1–6^, and the design freedom afforded by the DLW technique has been increasingly leveraged for integrating high resolution 3D structures within silicon^7^, glass^8,9^, and polydimethylsiloxane (PDMS)^10–13^ microfluidic systems. In-channel DLW has been employed to realize a variety of micromechanical^7,12,13^ and microfluidic^8,10^ components, 3D topological features^9^, and biomolecule-based 3D structures^11^ within pre-fabricated microchannels.

The DLW technique has also been used to integrate 3D microstructures into microfluidic devices fabricated from thermoplastics, which offer dimensional stability approaching that of glass or silicon while being amenable to replication-based fabrication for low cost manufacture. Recently, Alsharhan et al. demonstrated DLW printing of a microfluidic valve within a cyclic olefin polymer (COP) thermoplastic device^14^, yielding a bellows type flow valve with a deformable gate structure as thin as 500 nm. Similarly, Mark et al. performed DLW within an injection-molded thermoplastic chip to form a woodpile-shaped porous network containing micropores as small as $5\,\upmu \hbox {m}$ for the study of dendritic cell migration^15^.

A challenge associated with printing DLW structures directly within microchannels is that the heterogeneous materials limit the interfacial strength between the patterned photoresist and mating microchannel surfaces. Achieving high bond strength is particularly important for DLW-based microfluidics, since the laser writing process enables the fabrication of fluidic paths with sub-micrometer cross-sectional dimensions and millimeter or centimeter length scales that demand high pressure gradients to support convective flow. Several strategies have been reported to increase the adhesion of DLW structures within PDMS-based devices, allowing the resulting devices to operate at higher pressures without interfacial failure. Sol–gel reactions induced by pretreating the PDMS surface with 3-aminopropyl triethoxysilane (APTES) can promote chemical bonding with the epoxy-based DLW resin^11,16^, yielding devices reported to withstand burst pressures as high as 75 kPa^11^. Alternatively, silane-based adhesive introduced through a gap between the print and the elastomer channel walls has been used to provide cohesive sealing of the photoresist/PDMS interface, enabling operating pressures up to 200 kPa^10^. However, the low elastic modulus of elastomers such as PDMS presents an inherent limit to the achievable robustness of the resin/substrate interface.

Unlike PDMS, thermoplastics are rigid materials that support high pressure microfluidic applications without significant deformation of the substrate. However, most thermoplastic materials offer low surface energy and lack chemical handles that may be used to promote bonding. Operating pressures up to 500 kPa have been reported for $10\,\upmu \hbox {m}$ thick barriers fabricated by DLW within native COP microchannels^14^. To further improve the bond strength and open the door to applications requiring higher operating pressures, chemical modification of the thermoplastic surfaces is necessary. Various thermoplastic surface modifications have been developed to increase the interfacial bond strength without degrading the dimensional fidelity of microstructures that is often observed with thermal or solvent-mediated bonding methods^17^. These approaches typically require an initial surface activation step using oxygen radicals from energetic sources such as oxygen plasma or UV-ozone to overcome the chemically inert nature of thermoplastic polymers through chain scission and surface oxidation, but these methods are limited by both the initial achievable surface energy and relaxation of the surface over time. As an alternative approach, polymer photografting can offer distinct advantages for thermoplastic surface attachment. Light-controlled polymerization using a photoinitiator is an organic photochemistry technique that can yield complex co-polymer structures with high controllability^18^, and offers a versatile solution for grafting polymers on polymeric substrates^19–23^. In this process, the photoinitiator plays essential roles not only in forming active sites for chemical reaction but also making the reaction switchable between “on” and “off” states.

Benzophenone (BP) is one of the most prominent photoinitiators for this application, owing to its exceptional photochemical properties including high quantum yield, highly stable triplet excited states, and importantly, the ability to initiate hydrogen abstraction reactions in the presence of a hydrogen donor, such as a polymer surface^19,22^. Leveraging these features, chemical grafting using BP has been successfully demonstrated for various heterogeneous polymer systems, for example, poly(acrylic acid) (PAA) and poly(methyl methacrylate) (PMMA) with low-density polyethylene films^19^, poly(ethylene glycol) methacrylate (PEGMA) with cyclic olefin copolymer (COC) substrates^20^, PEGMA with methacrylate-based porous monoliths^21^, polystyrene with polypropylene (PP) substrates^22^, and PAA with PP membranes^23^.

While photografting is an effective strategy for achieving covalent bonds between mating polymers, its application to photoresists such as those employed in DLW is limited by the fact that the UV energy used to generate radicals at the bond surface also interacts with photoinitiators in the resist itself, resulting in unwanted bulk polymerization and negating the ability to pattern features within the material. However, we posited that the DLW laser may be harnessed to selectively activate the surface during laser writing, allowing localized photografting to occur during the pattern formation process. In this study, we demonstrate the use of polymer photografting during DLW to promote covalent bonding between the patterned photoresist resin and sealed thermoplastic COP microchannel surfaces. In this approach, a photoinitiator is first used to render the polymer microchannel surfaces photosensitive prior to DLW, followed by localized covalent bond formation between the resin and COP by utilizing the focused laser from the DLW printer to link the materials during the printing process. This unique approach is studied using COP surfaces pretreated with BP at varying concentrations and UV doses to identify optimal conditions for linking resin with the COP surface. The impact of feature thickness and use of mechanical interlocking on interfacial bond strength is further studied, and the use of different photoinitiators is also explored as a path toward enhancing the in situ photografting process.

## Results and discussion

### Sequential photoinitiation for covalent bond formation

**Figure 1:**
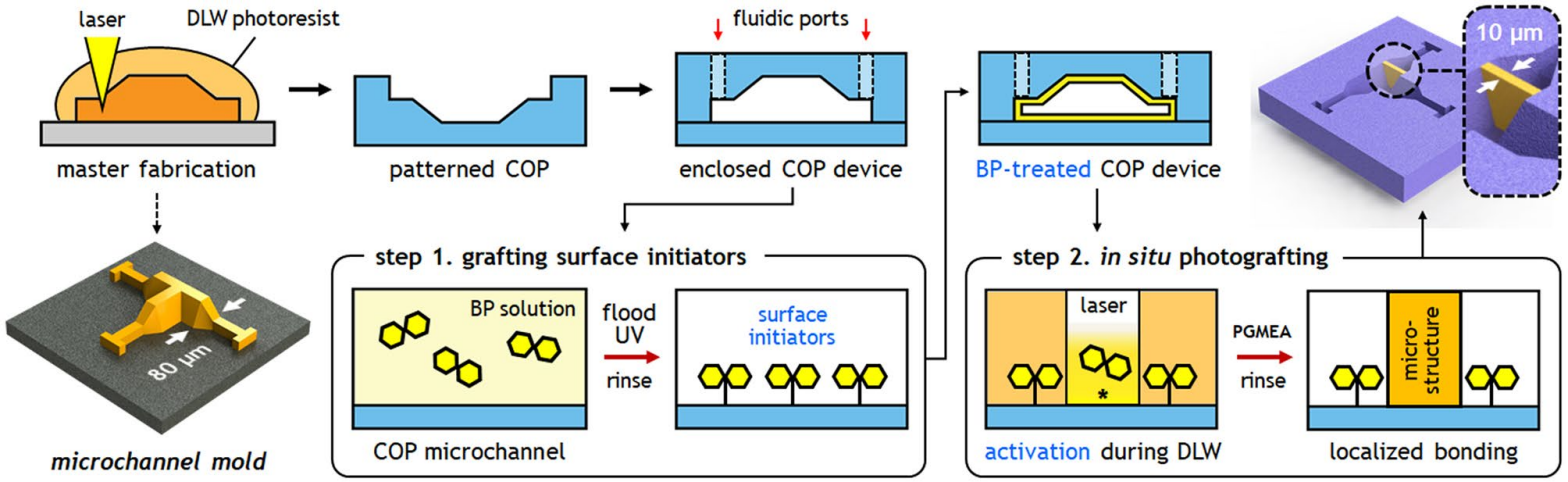
Two-step in situ photografting process. A microchannel mold with angled sidewalls formed via DLW is used to pattern microchannels in a COP substrate by hot embossing. The COP substrate is bonded with a thin COP film to enclose the fluidic path. Surface initiators are immobilized on the COP microchannel surface by filling the channel with BP solution, followed by UV flood exposure to promote the conversion of BP molecules into surface-bound photoinitiators (step 1). The channel is then filled with photoresist prior to direct laser writing. When the path of the DLW laser used to pattern integrated 3D microstructures coincides with the COP surface, the immobilized surface initiators are locally activated, resulting in immediate reactions with the photoresist polymer undergoing bulk polymerization (step 2). The final DLW microstructure is covalently attached to the COP surface.

**Figure 2:**
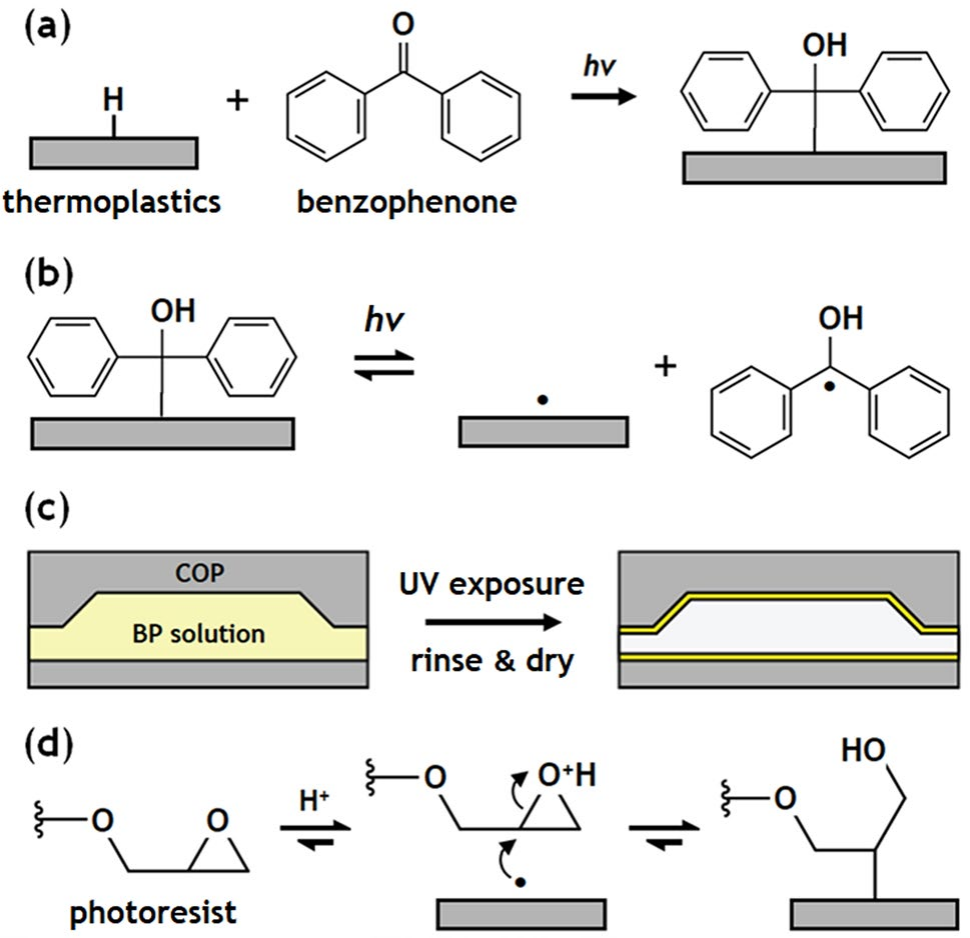
Chemical modification of thermoplastic surface using BP. (**a**) BP is converted into a semi-benzopinacol or its radical derivatives under UV irradiation, and reacts with thermoplastic polymer which serves as a proton donor. (**b**) The BP-treated thermoplastic surface may then be photoactivated for covalent bond formation between the surface and an exogenous material upon UV irradiation. (**c**) A COP microchannel filled with BP solution, and exposed to UV, rinsed, and dried, yielding a photoactive surface before introducing a photoresist in preparation for DLW. (**d**) Covalent bond formation scenario for the epoxy-based resin. When the laser path coincides with the microchannel surface, free radicals are formed on the COP surface that immediately react with the epoxide ring of the resin, yielding a covalent bond between the resin and thermoplastic surface.

Covalent bond formation between the photosensitive DLW resin and a COP substrate is achieved via a two-step process (Fig. 1): (i) grafting surface initiators on the COP surface, and (ii) photoactivating surface initiators using the DLW tool during printing. The first step takes advantage of the photocleavage mechanism of BP. Photoinitiators following Norrish type II photodecomposition, including BP, preferentially undergo hydrogen abstraction in the presence of proton donors under UV irradiation^18,19,23–26^. When BP is exposed to UV light on a COP substrate in the absence of monomer, it produces a photosensitive COP surface with chemically bound semi-benzopinacol surface initiators (Fig. 2a), while the surface initiator remains dormant until another photoabsorption event occurs (Fig. 2b)^20^. This principle provides strong advantages for thermoplastic-based systems. First, it offers a fundamentally different approach to conventional chemical modification methods for thermoplastics, which primarily rely on oxygen radicals to increase surface energy via oxidation and scission of polymer chains^17^. Second, many thermoplastic materials commonly used for microfluidic chips exhibit good UV transparency, down to 240 nm wavelength for COC and COP, and 260–300 nm for PMMA and polycarbonate^17,27,28^, thereby allowing photoabsorption within a sealed device to be achieved with minimal energy loss from the substrate material. Finally, for application to in-channel DLW, the ability to leverage the writing laser for the final crosslinking step during layer-by-layer patterning ensures that high optical energy density is available for photografting without losses due to optical absorption by the polymerized photoresist.

The concentration of photoresist solution and the UV dose are two primary parameters that determine the effectiveness of surface conversion. Generally, higher concentration of photoinitiator and higher UV dose promote denser surface initiator sites. However, screening effects can occur at high photoinitiator concentration due to high UV absorbance^20,22^, and it has been reported that varying exposure time at a fixed concentration is a more effective strategy to control the final density of surface initiator^21^. In this study, preliminary tests were performed for BP concentrations ranging from 0.01 to 10 wt% in methanol. At BP concentrations below 0.1 wt%, interfacial bond strength was comparable to untreated controls, while above 5.0 wt% saturation with increasing UV dose was observed, presumably due to increased screening from the bulk BP solution. Furthermore, at BP concentrations above 1 wt%, device failure was dominated by delamination of the COP/COP bonding interface rather than failure of the bond between the DLW barrier and COP surface, making it difficult to deconvolute the impact of BP concentration on covalent bond formation during in situ photografting. Thus, 0.1 wt% and 1.0 wt% concentrations were selected for further study. In all experiments, devices were inverted halfway through the UV exposure period to minimize screening effects from the bulk BP solution.

The second photografting step utilizes the laser of the DLW system to locally activate surface radicals and subsequently trigger grafting of the resin polymers to the COP surface. When the laser is focused at the COP/resin interface during printing, two photon absorption provides sufficient energy to transform a surface activator into a semi-benzopinacol radical and a surface radical. The surface radical then can react to a telechelic polymer in the channel, yielding covalently grafted resin polymer on the COP surface. The surface radical can react with different types of resin whether its primary mechanism is free radical polymerization or cationic polymerization, such as IP-L or SU-8 photoresist, respectively (Fig. 2d). While an additional radical released from the surface can terminate ongoing polymerization via recombination, it is less likely to initiate non-grafting polymerization due to steric hindrance caused by the two benzyl rings of the semi-benzopinacol radical^18,22^. Thus, the bulk photoresist continues to undergo the desired two-photon polymerization to realize 3-dimensional pattern formation while surface radicals are activated to immediately form covalent bonds with adjacent photoresist when the laser path coincides with the photosensitive COP surface.

### Burst pressure characterization

**Figure 3:**
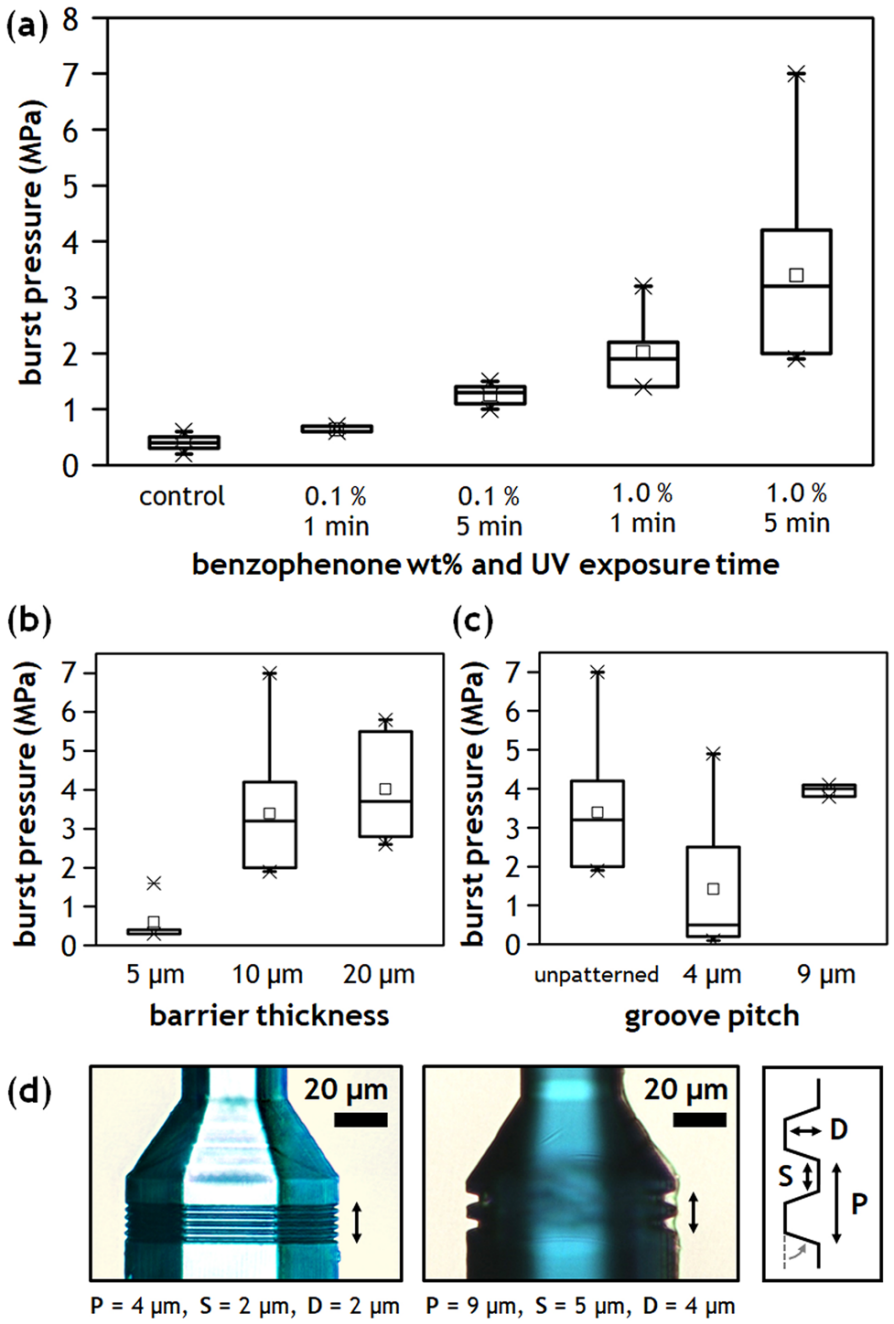
(**a**) Burst pressure measured across 10 $\upmu$m-thick in situ barriers printed on COP surfaces treated with varying BP concentrations and UV irradiation time. (**b**) Variation in burst pressure for different barrier thicknesses (1.0 wt% BP, 5 min UV) (**c**) Impact of surface topography on burst pressure using surfaces with trapezoidal cross section grooves (10 $\upmu$m-thick barriers, 1.0 wt% BP, 5 min UV). (**d**) Images of the grooved COP microchannels in (**c**), with selected geometries for groove pitch (P), spacing (S), and depth (D). Base angle of the trapezoidal cross section is fixed at 70$^\circ$.

The in situ photografting process promotes covalent bonds between the DLW resin and the COP surface. A straightforward strategy to improve bond strength is to increase the number of available reaction sites at the resin/COP interface by increasing either the bulk concentration of photoinitiator or the energy provided for the grafting reactions. For the first step of the sequential activation process, both parameters are readily modified. To this end, Fig. 3a presents the burst pressure measured across a 10 $\upmu$m-thick barrier printed within COP microchannels treated with varying BP concentrations and UV exposure times. In the absence of BP during the first step (control), the burst pressure was measured as 0.40 ± 0.14 MPa, in agreement with previously reported results for in-channel DLW using COP substrates^14^. The failure mode for this set of barriers was clean detachment from the COP surface (Supplementary Fig. S1a), confirming that barrier strength is dominated by the heterogeneous material interface. As seen in Fig. 3a, the BP concentration and UV exposure time both impact the resulting burst pressure, presumably by changing the activator site density. In the case of 0.1 wt% BP, burst pressures were measured 0.63 ± 0.06 MPa and 1.26 ± 0.21 MPa, for 1 min and 5 min UV exposure, respectively. The corresponding burst pressures were further increased to 2.02 ± 0.74 MPa and 3.39 ± 1.57 MPa for the case of 1.0 wt% BP. Overall, the initial BP concentration was found to have a more significant impact on burst pressure than UV exposure time, although both parameters play an important role.

**Figure 4:**
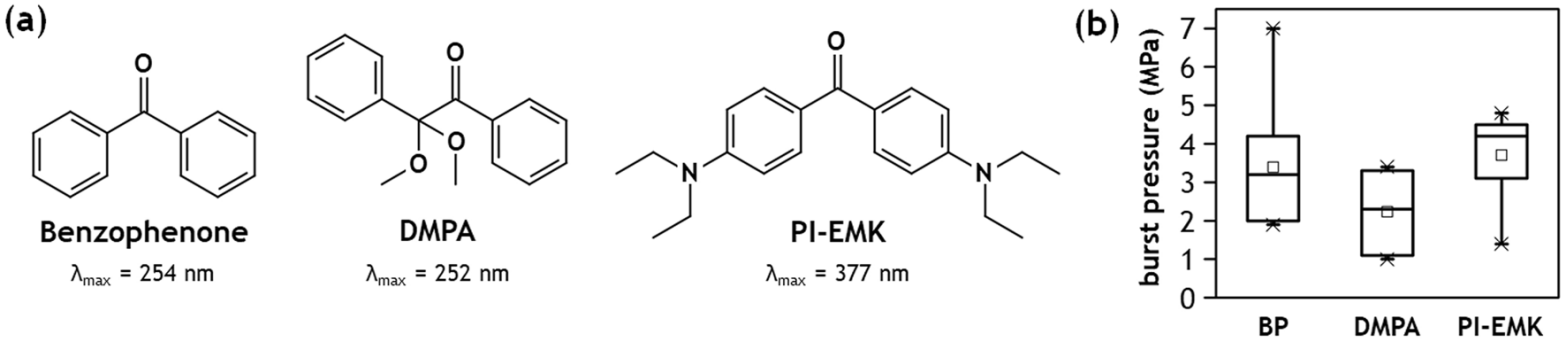
Comparison of photoinitiator performance. (**a**) Chemical structure of photoinitiators with the maximum absorption wavelength, and (**b**) burst pressure measured across a 10 $\upmu$m-thick barrier, printed on surfaces treated with the same mol% of each photoinitiator equivalent to 1.0 wt% BP (n $\ge$ 6 for all cases).

Increasing the contact area of the DLW print on the COP surface is another way to increase bond strength. Figure 3b shows the dependency of burst pressure on contact area, demonstrated by printing barriers of 5 $\upmu$m, 10 $\upmu$m, or 20 $\upmu$m thickness within COP microchannels treated with 1.0 wt% BP solution and 5 min UV irradiation. As anticipated, burst pressure increased with contact area, with measured values increasing 0.60 ± 0.56 MPa to 4.02 ± 1.36 MPa when increasing barrier thickness from 5 $\upmu$m to 20 $\upmu$m.

Pattering grooves in the substrate to anchor the printed structure can also serve to aid fluidic sealing^10^. This topological feature can improve the integrity of the interface by providing larger surface area for contact as well as by mechanically interlocking the mating layers. The efficacy of trapezoidal grooves with either coarse or fine features was explored (Fig. 3d). In each case, grooves were formed within a 20 $\upmu$m long section of the channel, and a 10 $\upmu$m-thick barrier was printed at the center of the grooved region. The measured burst pressure increased to 3.97 ± 0.15 MPa for devices printed on coarse grooves (Supplementary Fig. S1b), but was dramatically decreased to 1.43 ± 2.00 MPa when printed on the fine groove (Fig. 3c). Failure from flow leakage without any observed deformation or dislocation of the barrier accounted for 75% of interfacial failures among all devices patterned with the fine grooves, indicating that the grooves can mechanically constrain the barriers even after significant failure of the interface has occurred. The relatively poor performance of the fine groove case may be due to increased optical scattering from the smaller anchor features during barrier printing.

It is important to note that the bond strength of two solvent-bonded COP layers varies by the type of solvent and the conditions of the bond process. While the burst pressure of two thick solvent-bonded COP substrates can be as high as 35 MPa^17^, the COP devices used for in-channel DLW require a thin (100 $\upmu$m) COP film for bonding to support high resolution optical access to the channel during printing. This film can deform at much lower pressures than a thicker COP layer, making it more prone to delamination. In our experiments, COP film rupture or delamination of the COP/COP bonding interface rather than resin/COP interface failure was often observed. The maximum pressure tolerated by a single DLW barrier without failure across all experiments was 7.0 MPa, while failure of the COP/COP bonding interface or direct rupture of the COP film was observed at an average pressure of only 4.6 MPa. Thus, it is likely that the effective burst pressure that can be achieved using the in situ photografting strategy can be further increased by improving the quality of the bonding interface for the microfluidic substrate itself.

### Photoinitiator selection

The photoinitiator employed for the in situ photografting process should ideally be selected to maximize the density of activation sites for covalent bond formation. The aptness of a particular photoinitiator depends on both the photodecomposition mechanism and photoabsorption properties of the compound. A common way to categorize photoinitiators is by their photodecomposition mechanism. Norrish type I photoinitiators generally undergo homolytic cleavage at the excited C–C bond adjacent to the carbonyl group ($\alpha$-carbon) to produce two free radical fragments^25,29^, while Norrish type II compounds such as BP generate a pair of radicals via abstraction of a proton from a synergist (proton donor)^19,21,25^. The photoabsorption characteristics of the photoinitiator also impact efficiency of the photochemical reactions. This is particularly important for the second step of in situ photografting process since the DLW laser dictates the optical energy available for photoinitiation.

Here, we explored both of these criteria using a pair of additional photoinitiators: 2,2-Dimethoxy-2-phenylacetophenone (DMPA) and 4,4’-Bis(diethylamino) benzophenone (PI-EMK), for comparison with BP (Fig. 4a). Unlike BP, DMPA is a Norrish type I photoinitiator, but with an absorption spectrum similar to BP. During the first step of UV flood exposure to graft surface initiators to the thermoplastic surfaces, both initiators are converted into free radicals, but conversion of the DMPA (type I) is expected to occur primarily in the bulk solution while conversion of the BP (type II) occurs preferentially near the COP surface, which serves as proton donor. Thereby, DMPA is expected to be less effective than BP at forming dense surface activation sites for a given equivalent mole fraction of photoinitiator. This hypothesis is consistent with our experimental measurements, presented in Fig. 4b, with an average burst pressure of 2.23 MPa for a 10 $\upmu$m-thick barrier printed within DMPA-treated COP microchannels, a 34% decrease compared with the BP experiments.

In contrast, PI-EMK is a Norrish type II photoinitiator like BP, but with optical absorption tailored to longer wavelength photons. The DLW laser used in this study emits photons at a center wavelength of 780 nm. Since the photoinitiators are nearly transparent outside the UV range^4,26^, activation can only occur through the absorption of multiple photons. From Planck’s equation, the energy of two photon absorption of identical photons emitted from the DLW laser is $5.09 \times 10^{-19}\,\hbox {J}$, which is equivalent energy of a single photon of 390 nm wavelength. Thus, it is anticipated that PI-EMK, with an absorption peak of $\lambda _{\mathrm{max}} = 377\,\hbox {nm}$, will exhibit higher photoactivation efficiency during DLW than either BP ($\lambda _{\mathrm{max}} = 254\,\hbox {nm}$) or DMPA ($\lambda _{\mathrm{max}} = 252\,\hbox {nm}$). As expected, higher burst pressures were observed for the devices prepared by in situ PI-EMK photografting, with a mean value of 3.70 MPa. While this corresponds to only a 9.1% increase over BP, it is notable that half of the tested PI-EMK devices failed by bulk substrate delamination rather than barrier failure, while only 10% of the BP devices exhibited this failure mode, suggesting an even greater enhancement to adhesive strength for the PI-EMK treated devices.

## Conclusion

The in situ photografting method offers a facile and effective approach to forming robust interfacial bonds between photoresist structures and thermoplastic surfaces during DLW. The first photoinitiation step yields a polymeric surface with switchable reactivity, offering a simple route to surface functionalization for a range of thermoplastics, while the second grafting step is fully integrated with the DLW workflow, forming localized covalent bonds between the photoresist and thermoplastic surface during laser writing. By taking advantage of the DLW laser itself for photografting, the technique avoids the key limitation of conventional flood exposure, namely bulk polymerization of the photoresist that would fully inhibit the printing process. The ability to form robust bonds supporting pressures as high as 7 MPa using in situ photografting opens new opportunities for DLW-enabled microfluidic and nanofluidic systems requiring high operating pressures. More broadly, the technique may be also find utility for high resolution grafting of other materials such as biological macromolecules to thermoplastic surfaces, or for activating the surfaces of the DLW-patterned structures themselves.

## Methods

### COP microchannel device fabrication

The overall process including COP device fabrication, surface preparation, and in situ photografting is described in Fig. 1. The microfluidic device consists of three straight channels connecting with a T-shaped chamber conjoining the channels at the chamber center. Each channel is 40 $\upmu$m wide, 40 $\upmu$m tall, and 1.5 mm long. Design of the T-shaped chamber includes a box of 240 $\upmu$m length, 60 $\upmu$m height, and 80 $\upmu$m width attached at its midpoint to a branch of 60 $\upmu$m long and 80 $\upmu$m wide. The upper edges of the channels and T-shaped chamber are chamfered by 20$^\circ$, yielding a trapezoidal cross section designed to minimize optical diffraction and dispersion at the vertical side walls during DLW^10,11,14^. The chamber is connected with each straight channel via loft interconnects. A grooved section was formed at a position starting 10 $\upmu$m from each loft with either coarse or fine grooves with trapezoidal cross section. The coarse grooves were designed with 9 $\upmu$m groove pitch, 5 $\upmu$m spacing, and 4 $\upmu$m cut depth, and the fine grooves have respective dimensions of 4 $\upmu$m, 2 $\upmu$m, and 2 $\upmu$m. Both designs employed a 70$^\circ$ base angle for the trapezoidal cross-section. A 3D model of the microchannel device was prepared using SolidWorks software, then converted into a writing language using the DeScribe job preparation program (Nanoscribe GmbH, Germany). Conversion parameters were 0.5 $\upmu$m as XY hatching distance and 1.0 $\upmu$m as Z-slicing distance.

The COP microchannel device was prepared by pattern transfer from a DLW mold printed using the Photonic Professional GT DLW system (Nanoscribe). A positive mold of the design was formed on an indium tin oxide (ITO)-coated glass substrate (25 mm $\times$ 25 mm $\times$ 0.7 mm; Nanoscribe) using the IP-S negative-tone two-photon polymerization (2PP) resin and a 25$\times$ objective lens operating in the dip-in laser lithography writing mode. DLW parameters were set to 100 mm/s laser scan speed and 100 mW laser power. Once printing was completed, the substrate was developed in a propylene glycol methyl ether acetate (PGMEA) bath for 8 min with gentle agitation, followed by thorough rinsing with isopropanol (IPA) and drying on a 65 $^\circ \hbox {C}$ hotplate for 30 min. Finally, the print was hard baked on a hot plate at 165 $^\circ \hbox {C}$ for 30 min and slowly cooled to room temperature.

**Figure 5:**
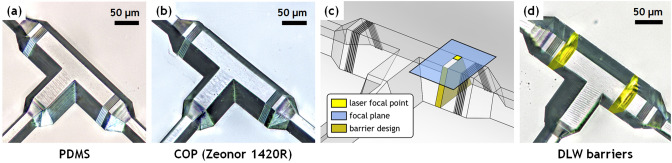
(**a**) PDMS positive mold used to pattern COP, and (**b**) negative COP substrate produced via hot embossing from the PDMS intermediate mold. (**c**) Illustration of the in-channel DLW process, with printing beginning at the top surface, furthest from the inverted objective lens, to avoid the need for focusing through any pre-printed structures. (**d**) A typical COP device with two DLW barriers. The barriers are false colored for visualization.

The inverse mold of the print was fabricated in 1020R Zeonor COP (Zeon Chemical, Louisville, KY) via hot embossing at 140 $^\circ \hbox {C}$ under 40 psi pressure using a benchtop hot press (12 ton PressPro; Dabpress, Shenzhen, China). To enhance pattern transfer from the IP-S master, the peak pressure was kept below 40 psi and the intervals between pressurization was approximately 2 min. While functional COP devices can be directly replicated from the IP-S master by hot embossing, the adhesion of IP-S resin on the ITO-coated substrate is generally insufficient to prevent pattern detachment over multiple hot embossing cycles. Thus, to improve production yield, a PDMS intermediate mold (Fig. 5a) was prepared from the COP inverse mold via soft lithography (1:10 wt ratio, Sylgard 184; Dow Chemical, Midland, MI). The cured PDMS mold was hard baked at 190 $^\circ \hbox {C}$ for 1 h before hot embossing using 1420R Zeonor COP pellets (Zeon Chemical) to produce microchannel-patterned COP substrates (Fig. 5b). Embossing was performed at 100 psi and $190\;^\circ \hbox {C}$ for 10 min, and the assembly was allowed to slowly cool to $80\;^{\circ }\hbox {C}$ over a period of approximately 20 min while maintaining a constant platen gap, thereby minimizing distortion of the molded part by allowing the elastic PDMS to recover its shape during cooling (Supplementary Fig. S2). The completed COP piece was trimmed to 2 cm $\times$ 2 cm using a table saw, and holes for fluidic interfacing were drilled manually at the end of each straight channels using a 650 $\upmu$m diameter drill bit.

The COP substrate was bonded with a piece of 100 $\upmu$m-thick Zeonor COP foil (Microfluidic ChipShop GmbH, Germany) by exposing the mating side of a COP foil to cyclohexane vapor at $35\;^{\circ }\hbox {C}$ for 105 s and subsequently compressing the assembly under 100 psi pressure for 5 min at room temperature. The completed device was placed in an oven at $65\;^{\circ }\hbox {C}$ overnight to remove residual solvent prior to surface treatment. Complete solvent removal was found to be critical to avoid bubble formation when the laser is focused near the bond interface during DLW.

### COP surface treatment using photoinitiators

Benzophenone (99%, Sigma Aldrich Inc., St. Louis, MO) stock solution was first prepared in methanol at 5 wt%, corresponding to 0.925 mol%, then further diluted with methanol for the concentration study. Stock solutions of 2,2-Dimethoxy-2-phenylacetophenone (DMPA) and 4,4’-Bis(diethylamino) benzophenone (PI-EMK) were prepared for the same mol% in methanol and acetone, respectively, then diluted with the same solvents.

To initially graft BP onto the COP microchannel surface, the BP solution was loaded into the microchannel and the fluidic ports were sealed to prevent solvent evaporation. Then, the device was irradiated (Fig. 2c) in a UV chamber (SunRay 400SM; Uvitron, West Springfield, MA) equipped with a 400 W UVA lamp for a specified time between 1–5 min, corresponding to an approximate total dose of between 1622–8109$\,\hbox {mJ/cm}^{2}$. To minimize potential screening effects from the photoinitiators, the device was turned over at half of the total exposure time. The device was then thoroughly rinsed with methanol and acetone, flushed with air, and stored in a light-protected environment prior to use.

### Barrier test structure printing

Barriers for burst pressure measurements were designed to fully block the microchannels, with cross-sectional dimensions expanded by an additional 5 $\upmu$m on each side and an additional 3 $\upmu$m on both the base and top, allowing to laser writing path to overlap into the bulk COP to ensure sufficient energy dose near the COP/resin interface for both resin polymerization and surface grafting. Conversion parameters for the fluidic barrier were 0.3 $\upmu$m and 0.5 $\upmu$m for XY hatching distance and Z-slicing distance, respectively.

Barriers were printed using the Photonics Professional GT DLW system equipped with a 63$\times$ objective lens in the oil immersion configuration. The COP device was prefilled with the IP-L 780 negative-tone 2PP resin using a plastic syringe, and mounted on the DLW tool with the COP film side of the device facing the objective lens. The laser path was defined to maximize the quality of the in-channel DLW^10,11,14^, with writing initiated at the farthest point from the objective lens and ending at the proximal surface to minimize optical interference from the polymerized resin (Fig. 5c). The laser scan speed was set to 10 mm/s, and the laser power was dynamically modified to compensate for optical absorption by the bulk photoresist^14^. For a 60 $\upmu$m tall microchannel, the laser power was adjusted between 50 mW, 40 mW, and 35 mW, for the upper, middle, and lower third of the channel depth, respectively. Higher laser power for the regions further from the laser source was employed to compensate for losses due to bulk prepolymer within the optical path, thereby improving optical dose uniformity throughout the channel volume. Higher optical power levels were not explored due to the potential for localized heating to generate solvent bubbles within the photoresist. After printing, the barrier was developed in a bath of PGMEA for 6 h on a shaker at 50 rpm, followed by IPA rinse and 6 h submersion in an IPA bath on a shaker. Despite the long development time imposed by the dead-end channel design, no delamination at the BP-treated COP surface was observed (Fig. 5d). The final devices were cleaned with a stream of nitrogen and dried in an oven at $65\;^{\circ }\hbox {C}$.

### Burst pressure characterization

Burst pressure was measured across individual barriers using an HPLC pump (PU-2089; Jasco Inc., Easton, MD). DI water was injected into the microchannel at 10 $\upmu$L/min while monitoring the back pressure at the inlet. Air trapped in the dead end channels was observed to compress during pumping, confirming a gas-tight seal prior to barrier failure at the device burst pressure. The burst pressure was determined from the maximum back pressure before a sudden drop was recorded. For properly printed devices, no fluid leakage was observed prior to catastrophic barrier failure. Barrier failure was confirmed under an optical microscope (LV 100; Nikon Instruments Inc., Melville, NY).

## Supplementary Information

Supplementary Information.
