# Supplementary material for: In situ photografting during direct laser writing in thermoplastic microchannels
Source: Sci Rep. 2021 May 26;11:10980. doi: 10.1038/s41598-021-90571-2 (PMC8155204; doi:10.1038/s41598-021-90571-2)
Supplement: Supplementary file 1 — Supplementary Information. [file 41598_2021_90571_MOESM1_ESM.pdf]

Supporting Information

**In Situ Photografting during Direct Laser Writing in Thermoplastic Microchannels**

*Jung Y. Han, Sarah Warshawsky, and Don L. DeVoe**

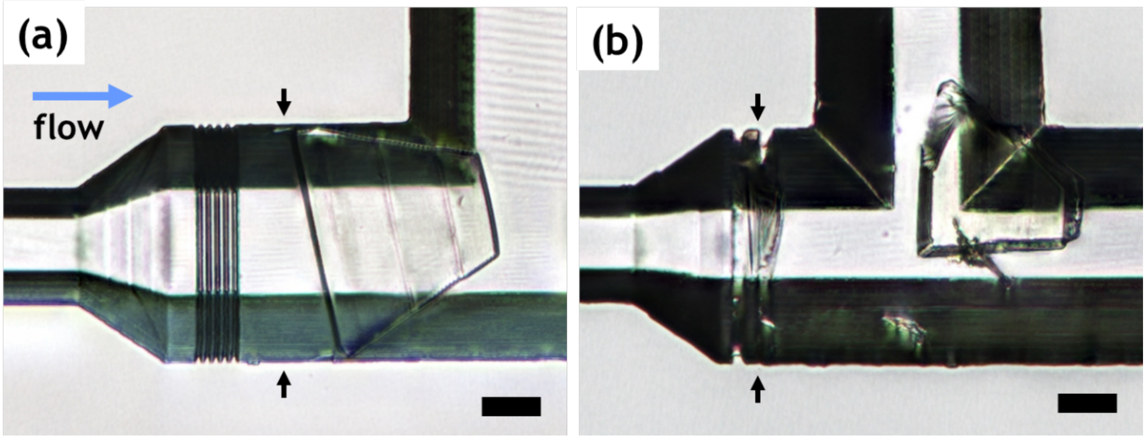


**Figure S1.** Failure scenarios during burst pressure tests. (a) Clean barrier detachment from a control device without surface modification, with failure observed at approximately 0.4 MPa inlet pressure. Clean separation of the barrier from the channel surface was observed. (b) Failure at 4.1 MPa inlet pressure for a device with *in situ* photografting, with barrier fragmentation observed. Original positions of the barriers are marked with arrows. All scale bars = 20 μm.

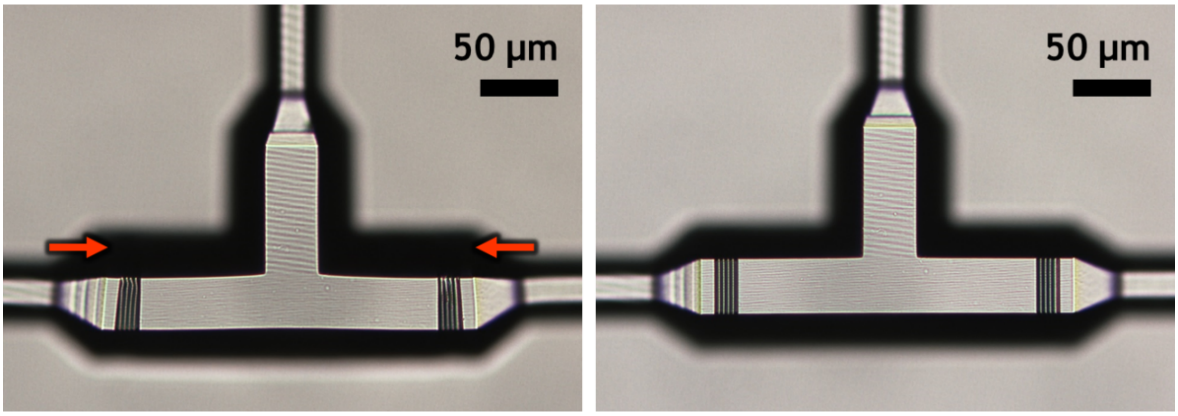


**Figure S2.** Effect of cooling rate on pattern fidelity for hot-embossed COP substrates made from a PDMS intermediate mold. (left) A microchannel-patterned COP substrate rapidly cooled to room temperature after hot embossing. (right) The same pattern transferred to a COP substrate when allowed to slowly cool after hot embossing.
